# Guest-mediated phase transitions in a flexible pillared-layered metal–organic framework under high-pressure†

**DOI:** 10.1039/d1sc03108b

**Published:** 2021-09-07

**Authors:** Gemma F. Turner, Scott C. McKellar, David R. Allan, Anthony K. Cheetham, Sebastian Henke, Stephen A. Moggach

**Affiliations:** School of Molecular Sciences, University of Western Australia Perth 6009 Western Australia Australia stephen.moggach@uwa.edu.au; EastChem School of Chemistry, University of Edinburgh Edinburgh EH9 3JW UK; Diamond Light Source, Harwell Science and Innovation Campus Didcot O11 ODE UK; Materials Research Laboratory, University of California Santa Barbara CA 93106 USA; Fakultät für Chemie und Chemische Biologie, Technische Universität Dortmund Dortmund 44227 Germany sebastian.henke@tu-dortmund.de

## Abstract

The guest-dependent flexibility of the pillared-layered metal–organic framework (MOF), Zn_2_bdc_2_dabco·*X*(guest), where guest = EtOH, DMF or benzene, has been examined by high-pressure single crystal X-ray diffraction. A pressure-induced structural phase transition is found for the EtOH- and DMF-included frameworks during compression in a hydrostatic medium of the guest species, which is dependent upon the nature and quantity of the guest in the channels. The EtOH-included material undergoes a phase transition from *P*4/*mmm* to *C*2/*m* at 0.69 GPa, which is accompanied by a change in the pore shape from square to rhombus *via* super-filling of the pores. The DMF-included material undergoes a guest-mediated phase transition from *I*4/*mcm* to *P*4/*mmm* at 0.33 GPa *via* disordering of the DMF guest. In contrast, the benzene-included framework features a structure with rhombus-shaped channels at ambient pressure and shows direct compression under hydrostatic pressure. These results demonstrate the large influence of guest molecules on the high-pressure phase behavior of flexible MOFs. Guest-mediated framework flexibility is useful for engineering MOFs with bespoke pore shapes and compressibility.

## Abstract 2

The guest-dependent flexibility of the pillared-layered metal–organic framework (MOF), Zn_2_bdc_2_dabco·*X*(guest), where guest = EtOH, DMF or benzene, has been examined by high-pressure single crystal X-ray diffraction.

## Graphical abstract

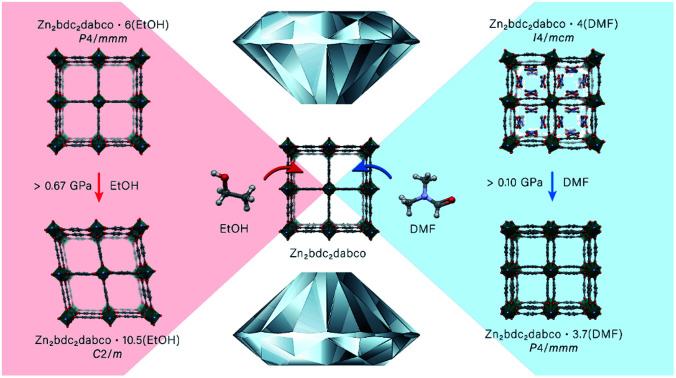

## Introduction

Flexible metal–organic frameworks (MOFs) may undergo dynamic and reversible structural changes under external stimuli, such as applied pressure or adsorption/desorption of guest.^1–5^ Structural flexibility is inherited from deformable metal coordination centres, conformational or rotational freedom of the organic linkers, structural defects, or flexible network topologies. Pronounced structural deformation may occur with retention of the framework crystallinity, allowing structural changes to be followed by *in situ* single crystal X-ray diffraction.^6^

High pressure is a convenient probe to examine the flexibility of MOFs, as it allows both the effect of direct pressure and pressure-induced guest adsorption on the structural response of the framework to be investigated.^7^ Presently, high-pressure X-ray crystallography using diamond anvil cell (DAC) apparatus has been used to survey the pressure-response of only ∼0.2% of the MOF structures deposited in the Cambridge Structural Database (CSD).^8^ In spite of the small number of MOFs studied, numerous generalised structural behaviours have been identified, such as ‘gate-opening’ linker rotations,^9–11^ ‘breathing’ of the pore volume,^12–14^ pore-shape changes,^10^ negative linear compressibility (NLC),^15–18^ polymorphic phase transitions,^19^ reversible or irreversible amorphisation,^20,21^ and even bond breakage,^22^ which are relevant to practical applications, including sensors,^23,24^ actuators,^1^ gas storage devices and filters,^25,26^ and drug-delivery.^27–29^ The pressure-response and mechanical properties of porous frameworks depends upon the nature and quantity of adsorbed guest, and is thus linked to the choice of pressure-transmitting medium (PTM), which, when small enough, can enter the framework pores during compression.^10,30^

The flexible response of a MOF to applied pressure can often be predicted based upon its local and topological structural features. Flexible secondary building units, such as multi-nuclear metal-oxo clusters, or metal-based paddlewheels, serve as hinges to facilitate deformation of the network. For guest-loaded MOFs, pressure-induced structural changes often depend upon host–guest interactions in the system, and are driven by stabilisation (or to minimise destabilisation) of these interactions at high-pressure.^31,32^

Guest-adsorption usually results with an increase in volume of the crystal, where the pore volume swells to accommodate the incoming guest.^30,33^ Extreme expansion or contraction of the pore volume upon guest adsorption and desorption corresponds to breathing.^12,13^ In other frameworks, such as ZIF-8,^9^ Sc_2_bdc_3_,^10^ and pillared-layered MOFs,^10^ pressure-induced guest adsorption prompts rotation of the organic linkers, which is associated with an increase in the vibrational entropy of the material.^34,35^ The occurrence and extent of the structural transition depends upon both the inherent flexibility of the framework and the host–guest interactions within the system.^1^

**Fig. 1:**
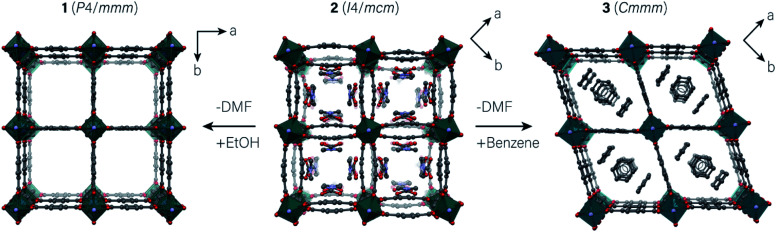
(Left to right) Square, rectangular and rhombus motifs of Zn_2_bdc_2_dabco·*X*(guest), where the guest is EtOH (**1**), DMF (**2**) or benzene (**3**), respectively. DMF and benzene guests are resolved in the crystal structures of **2** and **3**, while EtOH molecules are heavily disordered and cannot be resolved in the structure of **1**. The guest-free framework Zn_2_bdc_2_dabco has a structure almost identical to **1**. C, N and O atoms are shown in grey, light blue and red. The coordination polyhedra of the Zn atoms are displayed in teal.

Pillared-layered MOFs are a sub-class of flexible framework materials that are the subject of numerous publications on account of their dynamic structure behaviour.^18,35–41^ These materials are composed of two-dimensional (2D) M_2_L_2_ (M = Zn, Co, Cu or Ni; L = linear dicarboxylate) grids, extended in the third dimension by neutral, N-donor ‘pillars’. The prototypical pillared-layered framework, Zn_2_bdc_2_dabco (bdc^2−^ = 1,4-benzenedicarboxylate; dabco = 1,4-diazabicylo[2.2.2]octane), is a highly flexible material that adopts various geometries of the {Zn_2_bdc_2_} grid, depending upon the guest species occupying its pores (Fig. 1).^42^ Zinc paddlewheel units serve as deformable nodes in the {Zn_2_bdc_2_} grid, which structurally respond to changes in the host–guest interactions in the system.

When synthesised from a solvent of *N*,*N*-dimethylformamide (DMF), the {Zn_2_bdc_2_} layers adopt a rectangular geometry, characterised by an unusual bending of the bdc^2−^ ligands and an associated twisting of the Zn paddlewheel (Fig. 1, centre). Upon removal of the DMF guest molecules by heating to form the evacuated framework, the bdc^2−^ ligands become linear, forming a perfect square {Zn_2_bdc_2_} grid (Fig. 1, left).^42^ A further unique framework distortion is observed upon inclusion of benzene in the channels, where the {Zn_2_bdc_2_} grid adopts a rhombus geometry, caused by changes in the O–Zn–O angles around the paddlewheel (Fig. 1, right). Counterintuitively, the framework contracts slightly upon uptake of benzene and expands upon its release.^42^

In each structure, the dabco pillars are disordered along the crystallographic four-fold axis (or two-fold axis in the case of the benzene-included material). The DMF and benzene guest molecule occupy well-defined positions in the framework pores. Upon solvent exchange of the DMF for ethanol (EtOH), the square {Zn_2_bdc_2_} motif of the evacuated framework is formed.^42^ The EtOH molecules are disordered in the pore and cannot be directly modelled in the crystal structure.

Previous studies have shown that the breathing behaviour in Zn_2_bdc_2_dabco and its functionalised derivatives can be both dramatic and tuneable.^18,35–41^ Adsorption of isopropanol in the framework pores at ambient pressure triggers a breathing effect, with initial contraction of the pore upon adsorption of 3 molecules of isopropanol, followed by re-opening of the pore upon further adsorption of 1.5 guest molecules.^39^ At the highest guest loading, the bdc^2−^ linkers rotated by 25.5°. The magnitude and mechanism of the breathing in Zn_2_bdc_2_dabco can be tuned by functionalisation of the bdc^2−^ linkers or modification of the metal centre.^18,38,41,43^ Alkoxy-functionalised derivatives of Zn_2_bdc_2_dabco undergo volumetric contractions down to 72% of the original volume upon removal of DMF, EtOH or CO_2_ guest.^38^ Modification of the pendant alkoxy chains on bdc^2−^ changes the pressure at which the breathing transition occurs, in addition to its hysteretic behaviour, by changing the host–guest and intra-framework interactions. Similarly, modification of the metal centre in M_2_(BME-bdc)_2_dabco (where BME = 2,5-bis(2-methoxyethoxy)-1,4-benzenedicarboxylate and M = Zn^2+^, Co^2+^, Ni^2+^ or Cu^2+^), alters the electronics of the BME-bdc^2-^ linkers, affecting the magnitude and transition enthalpy of the pressure-induced and CO_2_-induced breathing.^18^ Combined empirical and theoretical analysis of the breathing mechanism in Zn_2_bdc_2_dabco and its Cu^2+^ analogue indicate a four-step transition from ‘closed-pore’ to ‘narrow-pore’ to ‘large-pore’ to ‘open-pore’ with increasing pressure, which is irreversible for Zn^2+^ reversible for Cu^2+^.^41^

Nanoindentation studies have shown that, in addition to directing structural changes in Zn_2_bdc_2_dabco, guest inclusion can also affect the mechanical properties of the framework, which are necessarily anisotropic by virtue of the two different ligand types present in the material.^36^ Anomalous thermal properties, such as uniaxial negative thermal expansion (NTE), have also been observed in Zn_2_bdc_2_dabco and its derivatives.^37^ The thermo-mechanical relationship between NTE and NLC in inherently anisotropic frameworks has been the subject of several studies showing the cooperative nature of the two phenomena, with occurrence of NTE serving as an indicator of the likelihood of NLC also occurring in the same direction.^44,45^ However, it has also been demonstrated that this inverse relationship is not always the case and NTE and NLC can, in fact, occur in perpendicular directions.^46^

The interplay between mechanical properties, structural behaviour and guest content make Zn_2_bdc_2_dabco an interesting material to study under high-pressure by single crystal X-ray diffraction. Here, we have investigated the structure of guest-loaded Zn_2_bdc_2_dabco·*X*(guest), where the guest species is EtOH (compound **1**, *X* = 6), DMF (compound **2**, *X* = 4) or benzene (compound **3**, *X* = 2). Whilst the guest-mediated flexibility of pillared-layered MOFs is recognised, there are only a handful of crystallographic studies that thoroughly characterise their structural response to both pressure and guest-adsorption.^2^

Together, **1**, **2** and **3** represent three unique but related frameworks with guest-dependent structures, adopting ‘square’, ‘rectangular’ and ‘rhombus’ {Zn_2_bdc_2_} motifs (or channel geometries), respectively. High-pressure single crystal X-ray diffraction data were collected on each framework within a diamond anvil cell (DAC) for the high-pressure environment,^47^ using the same guest present in the pores of each structure as the pressure-transmitting medium, to probe the effect of ‘super-filling’ of the framework under hydrostatic pressure. The effects of direct compression were also examined by using a non-penetrating pressure-transmitting medium of Fluorinert® FC-70 oil (perfluorotripentylamine).

The flexibility of Zn_2_bdc_2_dabco is found to depend on the strength of the host–guest interactions within the framework, which affect the geometry of the Zn paddlewheels and the shape of the channel walls (Zn-bdc-Zn). Pressure-induced uptake of guest into the channels prompts pore-shape modification, either by ‘super-filling’ of the channels with guest or by guest-mediated disordering of the ordered guest. The presence of guest within the pores is also found to impart NLC behaviour under pressure. This study showcases the structural versatility of a pillared-layered framework to guest and pressure, which is important to designing MOFs with bespoke functional and mechanical properties.

## Results and discussion

### Structure of Zn_2_bdc_2_dabco·6(EtOH) under pressure

Framework **1** was synthesised by post-synthetic guest exchange of the as-synthesised framework, **2**. Under ambient conditions, **1** is isostructural with the evacuated framework, Zn_2_bdc_2_dabco, crystallising in the space group *P*4/*mmm* with EtOH contained within the framework channels. According to thermogravimetric analysis (TGA), **1** contains ∼6 EtOH molecules per formula unit (ESI S3†). The disordered guest molecules were treated in the crystallographic model using the PLATON SQUEEZE algorithm,^48^ which gave a calculated electron density corresponding to 3.9 EtOH molecules per formula unit (Table 1 and ESI S2†). Whilst the guest content output from SQUEEZE is slightly inaccurate, it allows the relative change in the guest contents to be reliably followed during compression of the framework.

**Table 1:** Crystallographic and structural data for **1** at ambient pressure and variable applied pressures in a pressure-transmitting medium of EtOH. Reduced pore volumes and contents are given

	Ambient	0.31 GPa	0.69 GPa	1.19 GPa	2.10 GPa
Space group	*P*4/*mmm*	*P*4/*mmm*	*P*4/*mmm*	*C*2/*m*	*C*2/*m*
*a* (Å)	10.91350(4)	10.98050(4)	10.98300(4)	14.4123(12)	14.177(3)
*b* (Å)	10.91350(4)	10.98050(4)	10.98300(4)	16.3757(13)	16.361(3)
*c* (Å)	9.62600(4)	9.72200(4)	9.27200(4)	9.7030(4)	9.6086(11)
*β* (°)	n/a	n/a	n/a	92.158(4)	92.168(10)
*V* (Å^3^)	1146.50(1)	1172.20(1)	1118.45(1)	2288.4(3)	2227.1(6)
Pore *V* (Å^3^)	695	719	720	716	617
EtOH per pore^a^	3.9	7.8	8.1	11.9	11.9
Zn1–N1 (Å)	2.0629(4)	2.060(2)	2.047 (2)	2.104(14)	2.082(18)
Zn1–O1 (Å)	2.0225(15)	2.036(8)	2.055(9)	2.082(9)	2.086(12)
Zn1–O2 (Å)	n/a	n/a	n/a	1.993(9)	1.971(12)
Zn-bdc^b^ (°)	180.0	180.0	180.0	−171.5(6)	−170.8(8)
Zn⋯Zn^c^ (Å)	2.9148(5)	2.9769(28)	2.9901(28)	2.9748(1)	2.9421(41)
Grid distortion^d^ (°)	90	90	90	77.5	77.0

a Determined by PLATON SQUEEZE.^48^

b Zn–O1/O2–C1–C2 torsional angle.

c Intra-paddlewheel distance.

d Internal angle of channel, taken as the angle between intersecting planes defined by the bdc^2−^ ligands.

The framework is perforated by large, square channels (*ca.* 7.5 × 7.5 Å^2^) running parallel to the dabco pillars along the crystallographic *c*-axis. Two smaller channels (*ca.* 4.5 Å in diameter) intersect the larger channel along the *a* and *b*-directions, generating three-dimensional porosity.

When compressed in a pressure-transmitting medium of Fluorinert® FC-70, which is too large to enter the framework pores, the crystal of **1** became amorphous at the initial loading pressure of 0.1 GPa, and so no further structural analysis could be performed. A small single crystal (∼0.05 × 0.15 × 0.15 mm^3^) was used during the compression experiments to ensure that the crystal was not crushed by the DAC, and so the amorphisation of **1** is assumed to result from the generation of stress in the framework structure. Pressure-induced amorphisation is common at relatively low pressures for MOFs when compressed in a non-penetrating medium, although structural resilience can be improved by functionalising the organic linker or by loading the framework with certain guest molecules.^1,12^ To determine whether the presence of EtOH in the pores influences the structural resilience of the framework under pressure, the guest-evacuated framework was compressed under identical conditions. The evacuated framework also became amorphous at 0.1 GPa in Fluorinert® FC-70, implying that EtOH offers little mechanical strength to the material.

The pressure-response of **1** is distinct, however, when EtOH is used as the pressure-transmitting medium. Pressure-induced intrusion of EtOH into the framework pores during compression increases the mechanical stability of the material, delaying onset of amorphisation to >2.10 GPa. The anisotropy of the framework yields an interesting structural response to compression, affording two distinct regions in the pressure regime, marked by a phase transition from tetragonal (*P*4/*mmm*) to monoclinic (*C*2/*m*) above 0.69 GPa. The phase transition is associated with a three-fold reduction in symmetry, leading to the formation of a twin (ESI S5†).

Upon initial loading of **1** in the pressure cell at 0.31 GPa, the unit cell volume increased by 25.70(1) Å^3^ (+2.24%) as the number of EtOH molecules in the framework channels increased by approximately 4 (Table 1). Volumetric expansion is typical for MOFs when compressed in a penetrating medium, as the medium is forced into the pores under pressure, causing the pores to enlarge to accommodate the incoming guest.

Further compression up to 0.69 GPa caused the unit cell to decrease in volume by 53.75(1) Å^3^ (−4.59%). Compression of the unit cell from ambient pressure to 0.69 GPa coincides with a decrease in the inter-layer distance between {Zn_2_bdc_2_} layers along the *c*-axis due to compression of the Zn–N bonds in the dabco pillars by 0.016(2) Å (−0.77%) (Table 1). Between ambient pressure and 0.69 GPa, the *a*- and *b*-axes exhibit subtle negative area compressibility, increasing in area by 1.5218(8) Å^2^ (+1.3%), corresponding to extension of the equatorial Zn–O bonds in the paddlewheel units by 0.033(9) Å (+1.61%), which results in lateral expansion of the {Zn_2_bdc_2_} grid.

**Fig. 2:**
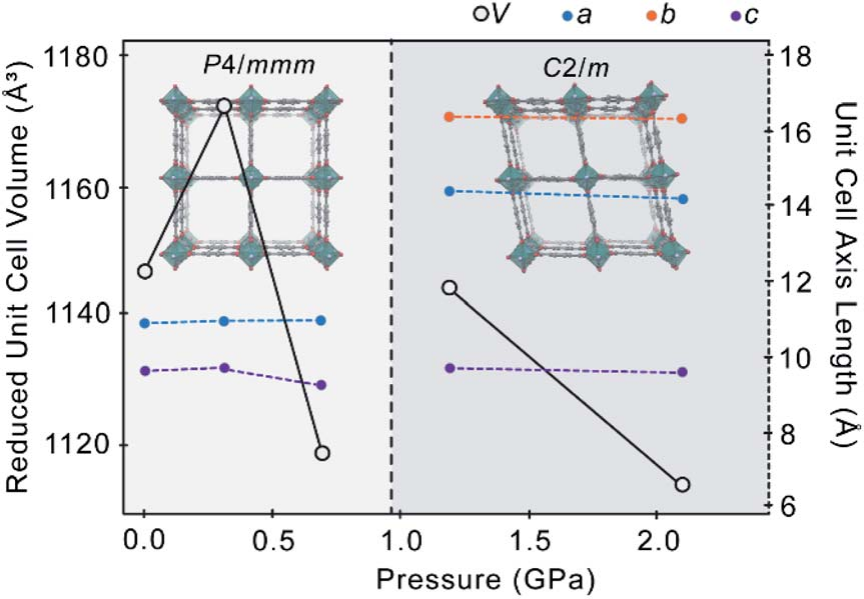
Reduced unit cell volume (white circles, black lines) and unit cell axis lengths (a – blue, b – orange, c – purple) of **1** as a function of pressure. The pressure-induced phase transition from a *P*4/*mmm* ‘square’ {Zn_2_bdc_2_} motif (0–0.69 GPa) to a *C*2/*m* ‘rhombus’ {Zn_2_bdc_2_} motif (>0.69 GPa) is caused by ‘super-filling’ of the framework pores with EtOH from the pressure-transmitting medium, and is indicated by a dashed line.

The EtOH content of the pores remains almost constant between 0.31 GPa and 0.69 GPa, indicating that the pores have reached their maximum capacity. Above a critical pressure at which the framework pores are full, MOFs typically undergo volumetric contraction as the guest molecules are squeezed out of the pores.^17^ Here, increasing the pressure further to 1.19 GPa caused the reduced unit cell volume to increase by 25.75(3) Å^3^, while the pore volume remained constant (Table 1). Rather than emptying the pores and compressing the native structure, the additional pressure drives a structural change to a more voluminous high-pressure phase, marked by a phase transition from *P*4/*mmm* to *C*2/*m* (Fig. 2).

The transition is characterised by a distortion of the square {Zn_2_bdc_2_} grid, whereby parallel layers of bdc^2−^ linkers undergo a slight lateral shift that breaks the *P*4/*mmm* symmetry and yields a new structure approaching the rhombus shape observed in the benzene-occupied material, **3**. The reduction in symmetry from *P*4/*mmm* to *C*2/*m* forms a twin (ESI S5†). There is a slight rotation of the Zn paddlewheels, such that the O–Zn–O angles deviate further from 90° and the Zn-bdc-Zn chains along the *a*- and *b*-axes are no longer perfectly linear, as highlighted by the ∠Zn1–O1–C1–C2 torsion angle of −171.5(6)° 1.19 GPa in the *C*2/*m* phase (Table 1). This significant flexibility around the Zn paddlewheel facilitates the structural change from square to rhombus channels without collapse of the framework. Flexible metal-centres are common in MOFs, such as in MIL-53, in which pressure-induced deformation of the O–M–O angles results in breathing between the ‘open-pore’ and ‘closed-pore’ structure^13^ or in AMU-1, which exhibits piezochromism due to generation of strain in the Co^2+^ coordination sphere during hydrostatic compression.^24^

Compression of the *C*2/*m* phase of **1** from 1.19 GPa to 2.10 GPa causes the unit cell volume to decrease by 61.0(7) Å^3^ (−2.7%) and the pore volume to decrease by 99 Å^3^ (−13.8%), with retention of the 11.9 molecules of EtOH. Unlike the *P*4/*mmm* phase, the *C*2/*m* phase does not show negative area compressibility or NLC, possibly due to the increased pressure being sufficient to cause direct compression of the framework in all directions. Direct compression of the framework under the additional pressure leads to an even more pronounced rhombic distortion of the square, with the angle between the planes defined by the pore walls (*i*.*e*. planes defined by bdc^2−^ ligands) decreasing from 90° at 0.69 GPa, to 77.5° at 1.19 GPa, and 77.0° at 2.10 GPa, with slight compression of Zn–N (Table 1). Above 2.1 GPa, the crystal became amorphous.

The poorer data quality caused by slight fracture of the crystal during the phase transition, coupled with low completeness of the diffraction data caused by a drop in the crystal symmetry, precluded accurate calculation of the pore content in the *C*2/*m* phase of **1**. However, from the increased unit cell volume on undergoing the phase transition, it is assumed that the EtOH content in the pores has increased (the EtOH content calculated from the high-pressure crystallographic data of low completeness is shown in Table 1.

Presumably, the change in the pore shape from square to rhombus results from ‘super-filling’ of the channels with EtOH, and the phase transition is a means by which the framework can accommodate the high guest loading. Since no similar transition was observed in **1** when Fluorinert® FC-70 was used as the pressure-transmitting medium, the phase transition must be mediated by EtOH inclusion at high-pressure.

A structural model of the guest EtOH molecules in **1** could not be resolved due to extensive dynamic disorder. However, it is clear that the phase transition is due to the quantity of EtOH forced inside the framework channels, possibly due to an increase in the cumulative strength of the host–guest interactions. The importance of host–guest interactions on governing the structure of a framework is evident from the structures of **1**, **2**, and **3** at ambient pressure, where different solvent-framework systems afford distinct {Zn_2_bdc_2_} motifs (Fig. 1). The square motif of framework **1** indicates that the EtOH-framework interactions are weak, and hence the phase transition only proceeds when the EtOH content of the pore exceeds a critical value where the host–guest interactions become significant.

### Structure of Zn_2_bdc_2_dabco·4(DMF) under pressure

Equivalent compression of the DMF-occupied framework, **2**, elicit a markedly different structural response than for the EtOH-occupied framework. At ambient pressure, the DMF molecules in **2** lie between the {Zn_2_bdc_2_} grids, adjacent to the dabco pillars, across a glide plane. At low temperatures, the guests are perfectly ordered in a *P*4/*ncc* structure,^49^ but at ambient temperature the oxygen atom of the DMF guest is disordered over two positions by mirror symmetry, inducing body-centring (*I*4/*mcm*). Host–guest interactions between the carbonyl of the DMF and CH_2_ groups of the dabco linkers appear to be responsible for the bending of the bdc^2−^ linkers. These interactions bring the DMF molecules into close proximity with the bdc^2−^ linkers, which bend to relieve steric strain with the other adjacent DMF molecule across the glide plane (O⋯O = 3.1(3)Å, CSD = ‘HIVSAI’).^36^ Hydrogen bonding interactions between the H-atom of bdc^2−^ and the O-atom of the DMF molecules stabilise the bent geometry (O⋯C = 3.8(2) Å, CSD = ‘HIVSAI’).^36^

**Fig. 3:**
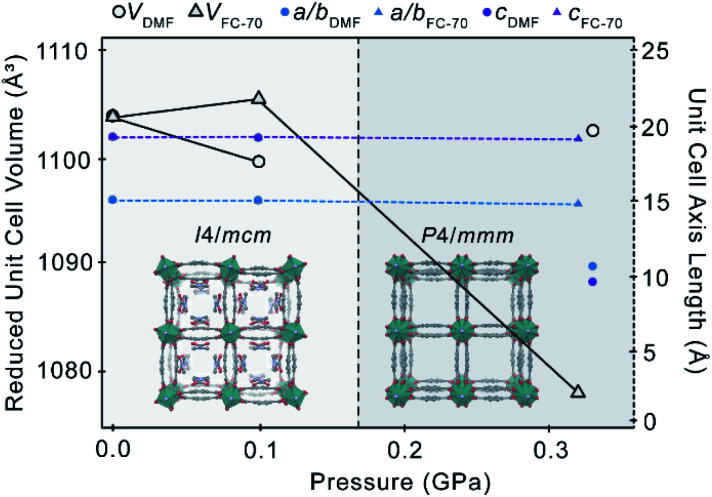
Reduced unit cell volume (white circles, triangles, black outline) and unit cell axes (a/b – blue, c – purple) of **2** as a function of pressure in a pressure-transmitting medium of either DMF (circles) or Fluorinert® FC-70 (triangles) . A phase transition from *I*4/*mcm* to *P*4/*mmm* occurs only in a medium of DMF by 0.33 GPa, and is marked by a dashed line.

Upon compression of **2** in a PTM of Fluorinert® FC-70 to 0.32 GPa, the unit cell volume decreases by 101.5(8) Å^3^ (−2.3%) (Table 2, Fig. 3), with retention of the *I*4/*mcm* symmetry. Above 0.32 GPa, crystallinity is lost. It is probable that the host–guest interactions impart a degree of mechanical stability to **2**, preventing immediate collapse of the framework, as was observed in **1** in a pressure-transmitting medium of Fluorinert® FC-70 (*ca.* 0.10 GPa), where no such significant host–guest interactions exist. Between ambient pressure and 0.32 GPa, the structure and DMF content of the framework are largely unchanged. The refined crystallographic occupancy of the DMF guest at 0.10 GPa and 0.32 GPa remains constant at 4 molecules per unit formula. Between ambient pressure and 0.10 GPa, the unit cell volume appears to increase, although the change here is small (0.16%).

**Table 2:** Crystallographic and structural data for **2** at ambient pressure and variable applied pressures in a pressure-transmitting medium of DMF or Fluorinert® FC-70. Reduced pore volumes and contents are given

	270^a^ K	0.10 GPa	0.33 GPa	0.10 GPa	0.32 GPa
PTM	n/a	DMF	DMF	Fluorinert® FC-70	Fluorinert® FC-70
Space group	*I*4/*mcm*	*I*4/*mcm*	*P*4/*mmm*	*I*4/*mcm*	*I*4/*mcm*
*a*, *b* (Å)	15.1208(10)	15.110(1)	10.6890(6)	15.130(18)	14.9647(9)
*c* (Å)	19.314(2)	19.2672(12)	9.6506(5)	19.32(4)	19.266(4)
*V* (Å^3^)	4415.9(6)	4398.9(6)	1102.63(10)	4423.0(12)	4314.4(11)
Pore *V* (Å^3^)	n/a	438	511	647	502
DMF per pore (refined, SQUEEZE)	4	4, 4.5	n/a, 4.5	4, 5.5	4, 4.5
Zn1–N1 (Å)	2.056(3)	2.051(8)	2.063(9)	2.05(3)	2.08(3)
Zn1–O1 (Å)	2.046(3)	2.045(6)	2.041(6)	2.054(7)	2.040(10)
Zn-bdc^b^ (°)	162.4(4)	164.5(6)	164.9(5)	163.5(5)	163.4(10)
Zn⋯Zn^c^ (Å)	2.951(1)	2.954(2)	2.9658(16)	2.984(12)	3.03(1)

a From CSD reference: ‘HIVSAI’ (deposit no. 986887)^36^ at ambient pressure and 270 K.

b Zn–O1/O2–C1–C2 torsional angle.

c Intra-paddlewheel distance.

Compression of **2** in a PTM of DMF promotes a guest-mediated structural response. The mechanical stability of the framework is unaffected by the DMF medium, becoming amorphous above 0.33 GPa (*ca.* >0.32 GPa in Fluorinert® FC-70), indicating that DMF does not appreciably infiltrate the channels during compression, in contrast to hyperfilling of **1** with EtOH. At the minimum loading pressure of 0.1 GPa, there is almost no change in the structure of **2**, nor the refined orientation or occupancy of the DMF guest. The unit cell is directly compressed by ∼17(1) Å^3^ (−0.4%) (Fig. 3).

As the pressure is increased to 0.33 GPa, **2** undergoes a phase transition from *I*4/*mcm* to *P*4/*mmm* – the same lattice symmetry as in the native Zn_2_bdc_2_dabco structure and in the EtOH-occupied framework, **1**. The phase transition is accompanied by disordering of the bdc^2−^ linkers across a mirror plane, which presumably destabilises the DMF adsorption sites through the introduction of steric hindrance, resulting in extensive dynamic disordering of the DMF guest. The DMF molecules can no longer be directly modelled in the crystal structure. Disordering of the DMF at 0.33 GPa breaks the mirror symmetry of the guest in the *I*4/*mcm* phase, resulting in the phase transition to *P*4/*mmm*. The bent geometry of the bdc^2−^ linkers forming the pores walls is retained during the phase transition, indicating that host–guest interactions are maintained.

The phase transition must be mediated by the DMF medium, since it does not occur in a PTM of Fluorinert® FC-70.

Guest-mediated phase transitions in porous materials are usually driven by pressure-induced adsorption of guest. According to the calculated content of DMF by SQUEEZE^48^ at 0.10 GPa and 0.33 GPa, the number of DMF molecules per channel remains constant at 4.5. However, the *I*4/*mcm* to *P*4/*mmm* transition is accompanied by an increase in the calculated pore volume by 73 Å^3^ (+16.7%) (Table 2), which is characteristic of guest adsorption. It is possible, therefore, that a small quantity of DMF from the PTM enters the pores at 0.33 GPa, which is insufficient to be reliably detected by SQUEEZE. Guest intrusion may then result in disordering of the DMF along the channel to relieve the steric contacts between adjacent adsorbents (O⋯O = 2.9(3) Å at 0.10 GPa). This is supported by the absence of any phase transition in **2** in Fluorinert® FC-70, which confirms this is not caused solely by pressure.

### Structure of Zn_2_bdc_2_dabco·3(benzene) under pressure

**Fig. 4:**
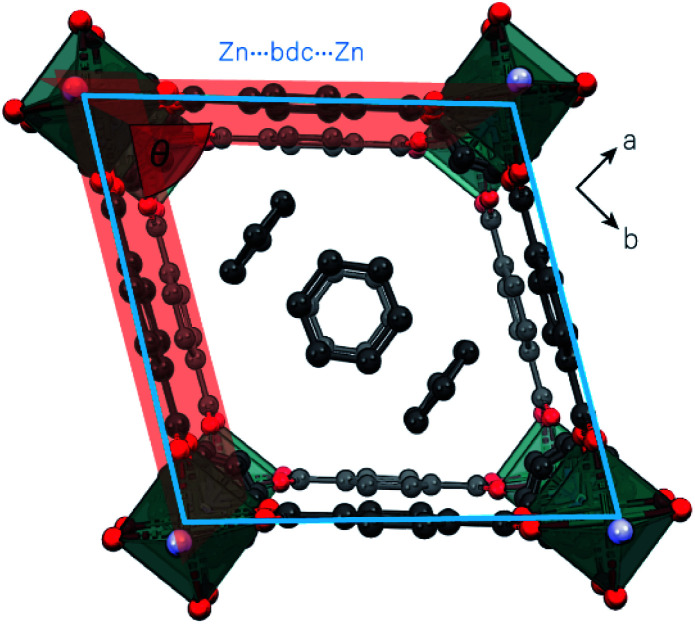
‘Rhombus’ pore of **3**, which becomes distorted under applied pressure, characterised by a decrease in *θ* (highlighted in red) and expansion of rhombus grid area (outline overlaid in blue). The grid size is determined by the Zn-bdc-Zn distance.

Unlike the EtOH and DMF occupied frameworks, **1** and **2**, the benzene-occupied framework, **3**, did not undergo any pressure-induced structural phase transitions. At ambient pressure, **3** adopts a rhombus motif of the {Zn_2_bdc_2_} grid (Fig. 1 and 4). Since ‘super-filling’ with EtOH at high-pressure is required to drive the structure of **1** towards the rhombus motif, we propose that the host–guest interactions in **3** are sufficiently greater than for **1** to produce the rhombus motif at ambient pressure.

Formation of the rhombus geometry of **3** is characterised by a decrease in the Zn-bdc torsional angle (∠Zn1–O1–C1–C2) from 180° in the native framework to 170.1(7)° in **3** (Table 3, *ca.* −171.7(5)° at 1.19 GPa for **1** in EtOH). The strong host–guest interactions are due to the benzene guest molecules, which lie in two well-defined, ordered positions and a third disordered position. One fully-occupied benzene sits in the centre of the pore and one is half-occupied in a perpendicular orientation near two corners of the {Zn_2_bdc_2_} grid, in close proximity to the dabco linker. The half-occupied guest is stabilised by CH⋯π contacts with the dabco pillar and shares CH⋯π interactions with the two closest bdc^2−^ linkers forming the corner of the channel, which induces the larger Zn-bdc torsion. The third benzene molecule is too disordered to be modelled.

**Table 3:** Crystallographic and structural data for **3** at variable temperature and variable applied pressures in a pressure-transmitting medium of benzene or Fluorinert® FC-70. Reduced pore volumes are given

	298^a^ K	0.15^b^ GPa	0.20^b^ GPa
PTM	n/a	Benzene	Fluorinert® FC-70
Space group	*Cmmm*	*Cmmm*	*Cmmm*
*a* (Å)	14.346(7)	13.6785(13)	13.575(3)
*b* (Å)	16.509(4)	16.9282(15)	16.994(3)
*c* (Å)	9.714(3)	9.6888(6)	9.6680(1)
*V* (Å^3^)	2300.6(14)	2243.5(3)	2230.3(7)
Zn1–N1 (Å)	2.077(4)	2.106(11)	2.074(19)
Zn1–O1 (Å)	2.028(3)	2.023(6)	2.002(11)
Zn-bdc^c^ (°)	173.5(2)	170.1(7)	175.4(5)
Zn⋯Zn^d^ (Å)	2.960(1)	2.957(2)	2.945(4)
Zn-bdc-Zn^e^ (Å)	10.936	10.8819(7)	10.875(2)
Rhombus area^f^ (Å^2^)	114.8	115.340(4)	117.53(4)
Grid distortion, *θ*^g^ (°)	78.5	71.9	72.2

a From CSD reference: ‘VURNED04’ (deposit no. 1426639)^40^ at ambient pressure.

b *T* = 298 K.

c Zn–O1/O2–C1–C2 torsional angle.

d Intra-paddlewheel distance.

e Pore wall length, *i.e.* the distance between centroids of Zn–Zn in neighbouring paddlewheels.

f Area depicted in Fig. 4, *A* = *pq*/2, where *p* and *q* are the distances between corners of the of the rhombus, taken from the Zn ions.

g Distortion of rhombus grid, taken as the angle between intersecting planes defined by the pore walls (bdc^2−^ linkers). Angle is illustrated in blue in Fig. 4.

It was only possible to collect X-ray diffraction data for **3** at a single pressure point in each of benzene and Fluorinert® FC-70 pressure-transmitting media before crystallinity was lost (ESI Table S3†). In both pressure-transmitting media, there was a slight decrease in the unit cell volume by 57.15(3) Å^3^ (−2.5%) and 70.35(7) Å^3^ (−3.1%) as the pressure was increased to 0.15 GPa or 0.20 GPa in benzene and Fluorinert® FC-70 media, respectively. Previous theoretical work^19^ hypothesised that the framework of **3** should contract upon uptake of benzene, rather than expand. It is possible, therefore, that the contraction in unit cell volume observed here is caused by entry of benzene into the framework pores from the pressure-transmitting medium under pressure. However, no additional ordered benzene molecules could be modelled within the pores, indicating that the contraction most likely results from direct compression of the framework.

Direct compression of **3** is also observed in a PTM of Fluorinert® FC-70. The compression of **3** in both benzene and Fluorinert® FC-70 hydrostatic media results in an expansion of the rhombus grid area grid by 0.54(4) Å^2^ (+0.47%) and 2.73(4) Å^2^ (+2.38%), respectively (Fig. 4, Table 3). Additionally, the grid becomes more distorted away from a square geometry at high-pressure, with the internal rhombus angle of the channel, taken as the angle between intersecting planes defined by the bdc^2−^ ligands, denoted as *θ*, decreasing from 78.5° at ambient pressure, to 71.9° (−8.35%) in benzene at 0.15 GPa, and 72.2° (−7.92%) in Fluorinert® FC-70 ay 0.20 GPa, respectively (Fig. 4, Table 3).

## Conclusions

In summary, we have examined the effect of guests on the structure and flexibility of the pillared-layered MOF, Zn_2_bdc_2_dabco·*X*(guest) at high-pressure, and have found that the nature and quantity of the guest species dictate that flexibility of the framework. Introduction of EtOH into the framework pores affords square-shaped channels, akin to the evacuated framework, indicating that the host–guest interactions are weak. Encouraging EtOH from the pressure-transmitting medium into the pores at elevated pressure increases the cumulative strength of the host–guest interactions in the system, prompting a phase transition above 0.69 GPa to a more voluminous phase with rhombus-shaped, ‘super-filled’ pores.

Compression of the as-synthesised MOF, which houses DMF in the pores and features bent bdc^2−^ linkers, draws a different response under pressure, undergoing a body-centred to primitive phase transition at 0.33 GPa when compressed in a pressure-transmitting medium of DMF. This transition is not seen when the DMF-occupied material is compressed in a non-penetrating pressure-transmitting medium, and so must be mediated by pressure-induced intrusion of the DMF medium into the framework channels. Occupation of the pores with benzene generates a framework with rhombus-shaped channels and alters the response of the MOF to pressure, causing direct compression of the material with NLC parallel to the long diagonal of the rhombus-shaped channels.

The ability to tune the structural response of MOFs to compression offers the potential to design host–guest systems with bespoke flexibility optimised for a particular application. Pressure and guest-induced changes in the pore-shape are particularly useful in storage applications, where the altered high-pressure pore-shape can increase the maximum storage capacity of the material *via* ‘super-filling’ of the framework pores. Similarly, tuneable mechanical properties, such as NLC, *via* guest-exchange offer the possibility to optimise the performance of MOFs as pressure sensors and actuators through materials engineering.

## Experimental

### Synthetic procedures

#### Synthesis of Zn_2_bdc_2_dabco·4(DMF) (**2**)

Zn(NO_3_)_2_·6H_2_O (250 mg, 0.84 mmol), 1,4-benzenedicarboxylic acid (140 mg, 0.84 mmol), and 1,4-diazabicyclo[2.2.2]octane (49 mg, 0.44 mmol) were suspended in 20 mL DMF. After stirring for 15 min the suspension was filtered and the resulting clear solution transferred into a Teflon lined steel autoclave (volume of 45 mL) and heated to 125 °C for 48 h. After cooling to room temperature, the crystals of **2** were filtered off and rinsed in 20 mL fresh DMF for 3 d. The DMF was exchanged against fresh solvent two times, after 24 h and 48 h. The crystals were kept in the DMF solution prior to further manipulation and analysis.

#### Synthesis of Zn_2_bdc_2_dabco·*X*(guest) (**1**, **3**)

Several crystals of **2** were filtered off and rinsed in 20 mL of either absolute EtOH or benzene for 3 d. The EtOH or benzene was exchanged against fresh solvent two times, after 24 h and 48 h. The crystals were kept in the EtOH (to form **1**) or benzene (to form **3**) solution prior to further manipulation and analysis.

#### Single crystal X-ray diffraction

##### Ambient conditions

Ambient pressure diffraction data for the EtOH-loaded MOF, **1**, were collected at room temperature on a Bruker APEX II diffractometer (Bruker 2002) using a Mo Kα X-ray source (*λ* = 0.7107 Å). Data were integrated using SAINT^50^ and absorption corrections were carried out using the program SADABS.^51^ Crystal structure refinements were carried out against |*F*|^2^ in CRYSTALS.^52^ The pore volume and solvent content of **1** were calculated using the SQUEEZE algorithm in PLATON,^48^ whilst the guest content of **2** and **3** were modelled directly in the structure model. All atoms, excluding those in the modelled solvent, were refined with anisotropic displacement parameters. The atomic positions of all non-hydrogen atoms were allowed to refine freely. Hydrogen atoms were placed geometrically and constrained to ride on their host atoms.

##### High-pressure

High-pressure diffraction experiments were performed in a modified miniature Merrill–Bassett diamond anvil cell^47^ (half-cell opening 40°), equipped with 600 μm culet Boehlar Almax diamonds, a tungsten gasket and tungsten carbide backing seats.^53^ A chip of ruby was used as the pressure calibrant *via* the ruby fluorescence method.^54^ Diffraction data for **1** and **3** were collected on a Bruker APEX II diffractometer using Mo Kα radiation. Diffraction data for **2** were collected using synchrotron radiation (*λ* = 0.48590 Å) at Diamond Light Source (UK). Data were collected in *ω*-scans in eight settings of 2*θ* and *φ* with a frame time and step size of one second and 0.3°, respectively (based a collection strategy described by Dawson *et al.*).^55^ Data were integrated in SAINT using dynamic masks to remove areas of the diffraction image that are shaded by the cell body. Absorption corrections for the body of the cell were applied using SHADE.^56^ Structure refinements were carried out against |*F*|^2^ in CRYSTALS,^52^ using the ambient pressure structures as starting coordinates. The pore volume and EtOH content of **1** were calculated and modelled using SQUEEZE in PLATON,^48^ although the output could not be trusted above 0.69 GPa due to fracturing of the crystal at higher pressures. Above 0.69 GPa, the crystal undergoes a phase transition, causing twinning of the crystal. The data collected at 1.19 GPa and 2.10 GPa were integrated as a twin, with the absorption correction carried out using Twinabs.^57^ Where possible, the solvent content of **2** and **3** were modelled directly, and were otherwise modelled using SQUEEZE. For **1**, all atoms were refined with anisotropic displacement parameters (ADPs) at ambient pressure, 0.31 GPa and 0.69 GPa, whilst only the Zn ions were refined anisotropically at 1.19 GPa and 2.10 GPa. For **2**, all atoms in the host framework were refined with ADPs, while the solvent DMF was refined with isotropic parameters. For **3**, all atoms were refined with ADPs, including the benzene guest molecules. Vibrational and thermal similarity restraints were applied to all non-metal atoms. Hydrogens were placed geometrically and constrained to ride on their host atom.

## Data availability

All crystallographic data have been deposited in the CSD. No other data is present.

## Author contributions

SCM, SH, SAM, DRA and AKC performed the experiments. SAM refined the crystal structures. SCM, SH, SAM, GFT and AKC analysed the results and prepared the manuscript.

## Conflicts of interest

There are no conflicts to declare.

## Supplementary Material

SC-012-D1SC03108B-s001

SC-012-D1SC03108B-s002
