# Supplementary material for: Guest-mediated phase transitions in a flexible pillared-layered metal–organic framework under high-pressure
Source: Chem Sci. 2021 Sep 7;12(41):13793–801. doi: 10.1039/d1sc03108b (PMC8549792; doi:10.1039/d1sc03108b)
Supplement: SC-012-D1SC03108B-s001 [file SC-012-D1SC03108B-s001.pdf]

Electronic Supplementary Material (ESI) for Chemical Science.
This journal is © The Royal Society of Chemistry 2021

Guest-Mediated Phase Transitions in a Flexible Pillared-Layered Metal-Organic Framework under High-Pressure

Gemma F. Turner,[a] Scott C. McKellar,[b] David R. Allan,[c] Anthony K. Cheetham,[d], Sebastian Henke,*[e] and Stephen A. Moggach*[a]

[a.] *School of Molecular Sciences, University of Western Australia, Perth, 6009, Western Australia, Australia*
[b.] *EastChem School of Chemistry, University of Edinburgh, Edinburgh, EH9 3JW, United Kingdom*
[c.] *Diamond Light Source, Harwell Science and Innovation Campus, Didcot, O11 ODE, United Kingdom*
[d.] *Materials Research Laboratory, University of California, Santa Barbara, CA 93106, USA*
[e.] *Fakultät für Chemie und Chemische Biologie, Technische Universität Dortmund, Dortmund, 44227, Germany*

Table of Contents

1. Crystallographic data ……………………………………………………………………………………1-3

2. Pore Volume and Contents ……………………………………………………………………………………4

3. Thermogravimetric Analysis ……………………………………………………………………………………5-6

4. FT-IR Spectroscopy……………………………………………………………………………………7

5. Twin Analysis ……………………………………………………………………………………8

1. Crystallographic Data

Supplementary Table S1. Experimental and crystallographic data for $Zn_2bdc_2dabco·X(EtOH)$ (**1**) in a PTM of EtOH at T = 298 K. Diffraction experiments were carried out on a Bruker Apex II diffractometer using a Mo X-ray source. Absorption was corrected for by multi-scan methods, SADABS (Siemens, 1996).

	**Ambient**	**0.31 GPa**	**0.69 GPa**	**1.19 GPa**	**2.10 GPa**
**Crystal data**					
Chemical formula	$(C_{22}H_{12}N_2O_8Zn_2)_n·3.9n(C_2H_6O)$	$(C_{22}H_{12}N_2O_8Zn_2)_n·7.8n(C_2H_6O)$	$(C_{22}H_{12}N_2O_8Zn_2)_n·8.1n(C_2H_6O)$	$(C_{22}H_{12}N_2O_8Zn_2)_n·11.9n(C_2H_6O)$	$(C_{22}H_{12}N_2O_8Zn_2)_n·11.9n(C_2H_6O)$
M_r	756.90	936.56	950.39	1125.4	1125.4
Crystal system, space group	Tetragonal, *P*4/*mmm*	Tetragonal, *P*4/*mmm*	Tetragonal, *P*4/*mmm*	Monoclinic, *C*2/*m*	Monoclinic, *C*2/*m*
a, *b*, *c* (Å)	10.91350(4), 10.91350(4), 9.62600(4)	10.98050(4), 10.98050(4), 9.72200(4)	10.98300(4), 10.98300(4), 9.27200(4)	14.4123(12), 16.3757(13), 9.7030(4)	14.177(3), 16.361(3), 9.6086(11)
α, β, γ (°)	90, 90, 90	90, 90, 90	90, 90, 90	90, 92.158(4), 90	90, 92.168(10), 90
V (Å^3)	1146.50(1)	1172.20(1)	1118.45(1)	2288.4(3)	2227.1(6)
Z	1	1	1	2	2
ρ (g cm^{-3})	1.097	1.329	1.348	1.633	1.678
Crystal size (mm)	0.05 × 0.15 × 0.15	0.05 × 0.15 × 0.15	0.05 × 0.15 × 0.15	0.05 × 0.15 × 0.15	0.05 × 0.15 × 0.15
**Data collection**					
T_{min}, T_{max}	0.78, 0.95	0.82, 0.95	0.83, 0.95	0.70, 0.95	0.71, 0.95
No. of measured, independent and observed [$I > 2.0σ(I)$] reflections	24471, 745, 718	4042, 386, 333	4233, 399, 346	8127, 1089, 824	2069, 769, 511
R_{int}	0.043	0.048	0.044	0.088	0.183
$θ_{max}$ (°)	26.4	25.0	25.6	23.3	20.8
$(\sin θ/λ)_{max}$ (Å^{-1})	0.626	0.595	0.609	0.556	0.501
**Refinement**					
$R[F^2 > 2σ(F^2)]$, $wR(F^2)$, S	0.024, 0.067, 1.04	0.076, 0.190, 1.02	0.076, 0.213, 1.04	0.133, 0.276, 1.51	0.177, 0.377, 1.30
No. of reflections	745	379	399	1038	747
No. of parameters	32	31	31	44	50
No. of restraints	73	78	69	121	86
$Δρ_{max}$, $Δρ_{min}$ (e Å^{-3})	0.36, −0.37	1.89, −1.17	1.26, −1.03	1.34, −1.38	1.46, −1.83

Supplementary Table S2. Experimental and crystallographic data for $Zn_2bdc_2dabco·X(DMF)$ (**2**) in a PTM of DMF or Fluorinert® FC-70 at T = 298 K. Diffraction experiments were carried out using synchrotron radiation ($λ$ = 0.48590 Å). Absorption was corrected for by multi-scan methods, *SADABS* (Siemens, 1996).

	**Ambient[a]**	**0.10 GPa**	**0.33 GPa**	**0.10 GPa**	**0.32 GPa**

**Crystal data**					
PTM	n/a	DMF	DMF	Fluorinert® FC-70	Fluorinert® FC-70
Chemical formula	$(C_{22}H_{12}N_2O_8Zn_2)_n{\cdot}4n(C_3H_7NO)$	$(C_{22}H_{12}N_2O_8Zn_2)_n{\cdot}4n(C_3H_7NO)$	$(C_{22}H_{12}N_2O_8Zn_2)_n{\cdot}4n(C_3H_7NO)$	$(C_{22}H_{12}N_2O_8Zn_2)_n{\cdot}4n(C_3H_7NO)$	$(C_{22}H_{12}N_2O_8Zn_2)_n{\cdot}4n(C_3H_7NO)$
M_r	855.49	863.55	863.55	863.55	863.55
Crystal system, space group	Tetragonal, *I*4/*mcm*	Tetragonal, *I*4/*mcm*	Tetragonal, *P*4/*mmm*	Tetragonal, *I*4/*mcm*	Tetragonal, *I*4/*mcm*
a, *c* (Å)	15.1208(10), 19.314(2)	15.110(1), 19.2672(12)	10.6890(6), 9.6506(5)	15.130(18), 19.32(4)	14.9647(9), 19.266(4)
V (Å^3)	4415.9(8)	4398.9(6)	1102.63(14)	4423(14)	4314.4(11)
Z	4	4	1	4	4
ρ (g cm^{-3})	0.830	1.15	1.11	1.14	1.17
Crystal size (mm)	0.25 x 0.16 x 0.13	0.05 × 0.15 × 0.15	0.05 × 0.15 × 0.15	0.05 × 0.15 × 0.15	0.05 × 0.15 × 0.15
**Data collection**					
T_{min}, T_{max}	0.04, 1.00	0.73, 0.94	0.58, 0.95	0.70, 0.94	0.57, 0.94
No. of measured, independent and observed [$I > 2.0\sigma(I)$] reflections	5445, 1416, 850	10160, 935, 575	5314, 600, 506	9425, 476, 386	5869, 301, 244
R_{int}	0.055	0.112	0.093	0.083	0.068
θ_{max} (°)	28.9	15.7	17.0	17.2	14.1
$(\sin \theta/\lambda)_{max}$ (Å^{-1})	0.680	0.625	0.625	0.610	0.500
**Refinement**					
$R[F^2 > 2\sigma(F^2)]$, $wR(F^2)$, S	0.065, 0.214, 1.11	0.101, 0.197, 0.98	0.117, 0.203, 1.01	0.179, 0.229, 1.08	0.193, 0.221, 1.03
No. of reflections	1416	904	557	428	261
No. of parameters	70	70	51	70	48
No. of restraints	18	165	167	165	79
$\Delta\rho_{max}$, $\Delta\rho_{min}$ (e Å^{-3})	0.66, -0.56	1.79, −1.28	1.74, −0.79	0.46, −0.37	0.65, −0.60

[a] From CSD reference: 'HIVSAI' (deposit no. 986887)[12a] at ambient pressure and 270 K

Supplementary Table S3. Experimental and crystallographic data for $Zn_2bdc_2dabco{\cdot}3$(Benzene) (**3**) in a PTM of benzene or Fluorinert® FC-70 at T = 298 K. Diffraction experiments were carried out on a Bruker Apex II using a Mo X-ray source. Absorption was corrected for by multi-scan methods, SADABS (Siemens, 1996).

	**0.15 GPa**	**0.20 GPa**
**Crystal data**		
PTM	Benzene	Fluorinert® FC-70
Chemical formula	$(C_{22}H_{12}N_2O_8Zn_2)_n{\cdot}2n(C_6H_6)$	$C_{22}H_{12}N_2O_8Zn_2{\cdot}2n(C_6H_6)$

M_r	733.44	733.44
Crystal system, space group	Orthorhombic, *Cmmm*	Orthorhombic, C*mmm*
a, *b*, *c* (Å)	13.6785 (13), 16.9282 (15), 9.6888 (8)	13.575 (3), 16.993 (3), 9.6680 (11)
V (Å^3)	2243.5 (3)	2230.3 (7)
Z	2	2
ρ (g cm^{-3})	1.11	1.12
Crystal size (mm)	0.05 × 0.15 × 0.15	0.05 × 0.15 × 0.15
**Data collection**		
T_{min}, T_{max}	0.58, 0.95	0.71, 0.95
No. of measured, independent and observed [$I > 2.0\sigma(I)$] reflections	3438, 682, 572	2039, 490, 351
R_{int}	0.068	0.082
θ_{max} (°)	23.3	22.0
$(\sin \theta/\lambda)_{max}$ (Å^{-1})	0.556	0.527
**Refinement**		
$R[F^2 > 2\sigma(F^2)]$, $wR(F^2)$, S	0.084, 0.210, 1.01	0.113, 0.287, 1.01
No. of reflections	667	485
No. of parameters	91	54
No. of restraints	185	144
$\Delta\rho_{max}$, $\Delta\rho_{min}$ (e Å^{-3})	0.68, −1.02	1.16, −0.97

2. Pore Volume and Guest Content

Supplementary Table S4. Ethanol content per pore of $Zn_2bdc_2dabco{\cdot}X(EtOH)$ (**1**) at variable pressure in a PTM of EtOH. Pore content and volume was calculated using the SQUEEZE algorithm in PLATON.[5]

	**Ambient**	**0.31 GPa**	**0.69 GPa**	**1.19 GPa**	**2.10 GPa**
Pore *V* (Å^3)	695	719	720	1431	1330
Red. pore *V* (Å^3)	695	719	720	716	665
e- count per unit cell	102	202	211	620	617
EtOH per pore	3.9	7.8	8.1	11.9	11.9

Supplementary Table S5. *N,N*-Dimethylformamide content per pore of $Zn_2bdc_2dabco \cdot X(DMF)$ (**2**) at variable pressure in a PTM of DMF or Fluorinert FC-70®. Pore content and volume was calculated using the SQUEEZE algorithm in PLATON.[5]

	**0.1 GPa**	**0.33 GPa**	**0.10 GPa**	**0.32 GPa**
PTM	DMF	DMF	FC-70	FC-70
Pore *V* (Å^3)	438	511	2586	502
Red. pore *V* (Å^3)	438	511	646.5	502
e- count	852	1011	1011	158
DMF per pore	5.5	4.5	6.5	4.1

3. Thermogravimetric Analysis

Thermogravimetric and differential thermal analysis (TG-DTA) was performed on a TA Instruments Q600 (sample weight approximately 5-10 mg) under nitrogen flow (flow rate 100 mL min^{-1}) in the temperature range from 25 °C to 600 °C with a heating rate of 10 °C min^{-1}. The solvated samples were taken out of the respective solvent (DMF, EtOH, or benzene), air-dried for approximately 1 h and placed in alumina crucibles for the measurement.

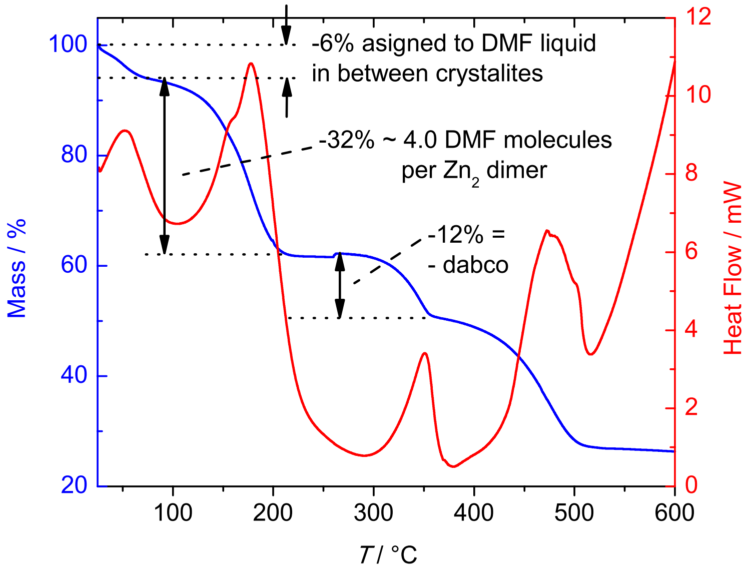

Figure S1: TG/DTA trace **2**. 32% weight loss up to a temperature of ~200 °C corresponds to the loss of 4.0 DMF molecules per formula unit of Zn_2bdc_2dabco. This is in excellent agreement with the single crystal XRD data. The second step in the range from 300 to 350 °C corresponds to the loss of dabco.

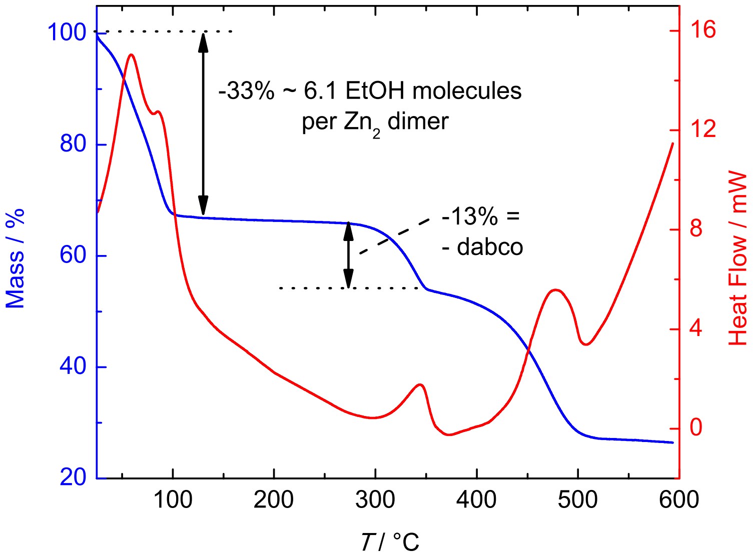

Figure S2: TG/DTA trace of **1**. 33% weight loss up to a temperature of ~100 °C corresponds to the loss of 6.1 EtOH molecules per formula unit of $Zn_2(bdc)_2dabco$. The second step in the range from 300 to 350 °C corresponds to the loss of dabco.

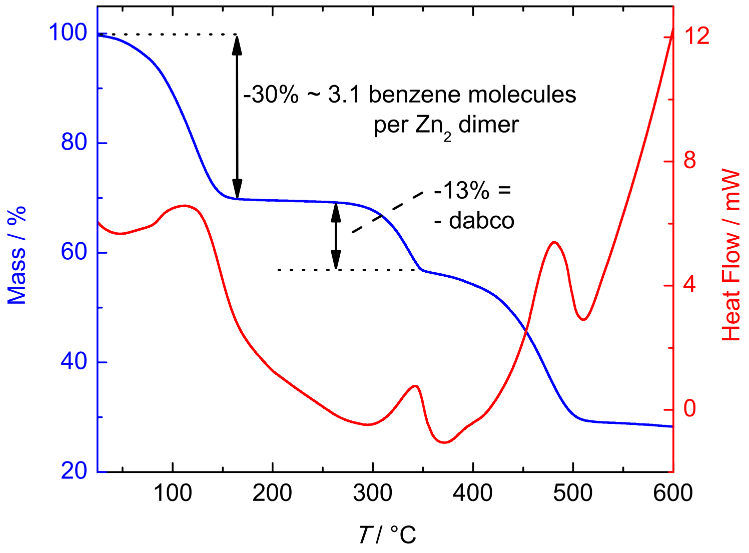

Figure S3: TG/DTA trace of **3**. 30% weight loss up to a temperature of ~150 °C corresponds to the loss of 3.1 benzene molecules per formula unit of Zn_2bdc_2dabco. Only two benzene molecules per formula unit could be located crystallographically, suggesting further disorder of the benzene guests. The second step in the range from 300 to 350 °C corresponds to the loss of dabco.

4. FT-IR spectroscopy

IR spectroscopy was carried out on air-dried samples on a Bruker Tensor 27 FT-IR spectrometer ($\tilde{\nu}$ = 520 cm^{-1} – 4000 cm^{-1}) in reflection mode using a diamond ATR (attenuated total reflectance) unit.

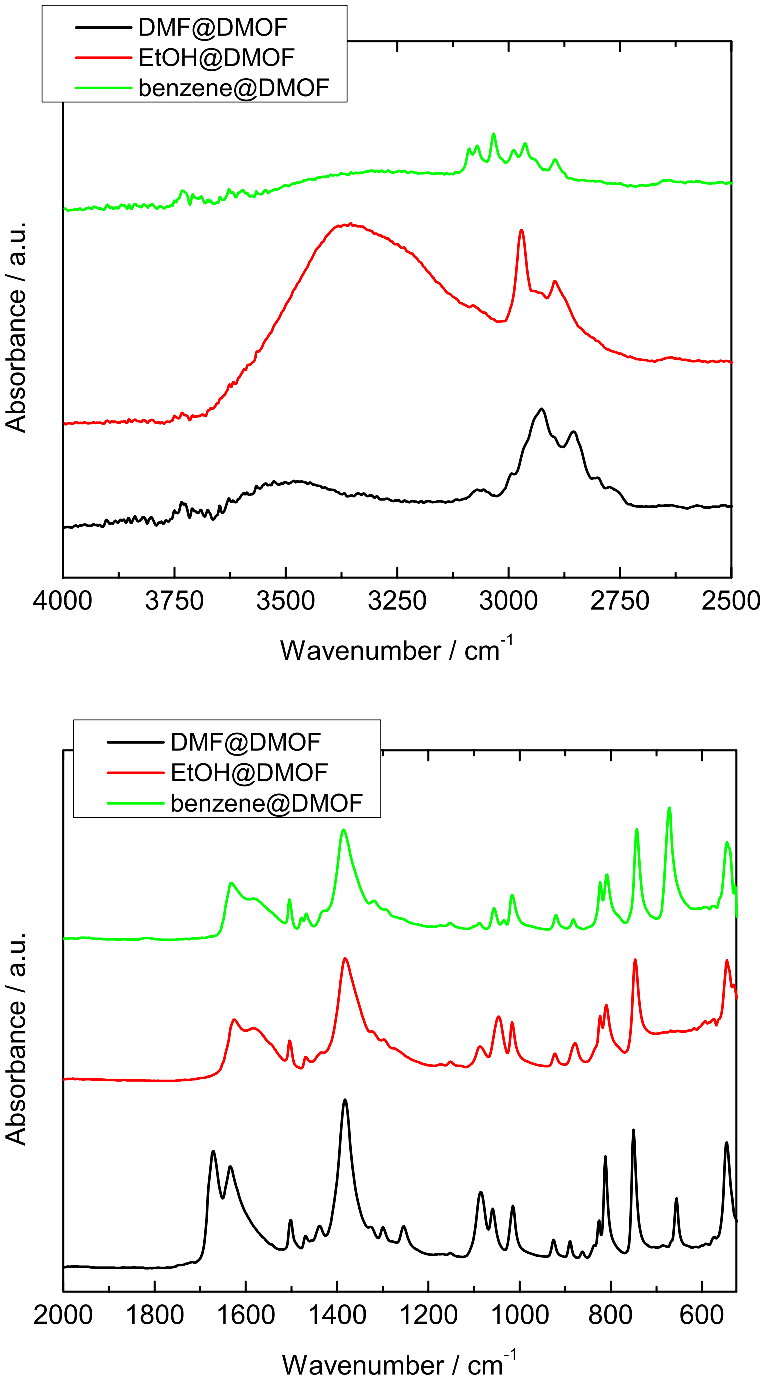

Figure S4: ATR-FTIR spectra of **1** (EtOH@Zn_2bdc_2dabco), **2** (DMF@Zn_2bdc_2dabco), and **3** (Benzene@Zn_2bdc_2dabco),. Top: Spectral region from 2500 to 4000 cm^{-1}. Bottom: Spectral region from 500 to 2000 cm^{-1}. The specific vibrational bands of the respective guest molecules are observed.

5 Twin Analysis

The structural phase transition of the ethanol-containing framework, **1**, during compression in a PTM of ethanol from *P*4/*mmm* to *C*2/*m* is accompanied by a three-fold reduction in symmetry, thereby inducing twinning of the crystal. Two twin laws were found using ROTAX in CRYSTALS, as listed below.

$$\text{Twin law 1 : } \begin{pmatrix} 1 & 0 & 0 \\ -0 & -1 & 0 \\ -0.051 & 0 & -1 \end{pmatrix} \times \begin{pmatrix} a \\ b \\ c \end{pmatrix} = \begin{pmatrix} a & 0 & 0 \\ -0 & -b & 0 \\ -0.051a & 0 & -c \end{pmatrix} \quad \text{(figure of merit = 8.171)}$$

$$\text{Twin law 2 : } \begin{pmatrix} 1 & 0 & 0.112 \\ 0 & -1 & 0 \\ 0 & 0 & -1 \end{pmatrix} \times \begin{pmatrix} a \\ b \\ c \end{pmatrix} = \begin{pmatrix} a & 0 & 0.112c \\ 0 & -b & 0 \\ 0 & 0 & -c \end{pmatrix} \quad \text{(figure of merit 8.709)}$$

Twining in framework, **1**, was also evidenced from synthetic precession images (Figure S5), where the separate components could be clearly resolved.

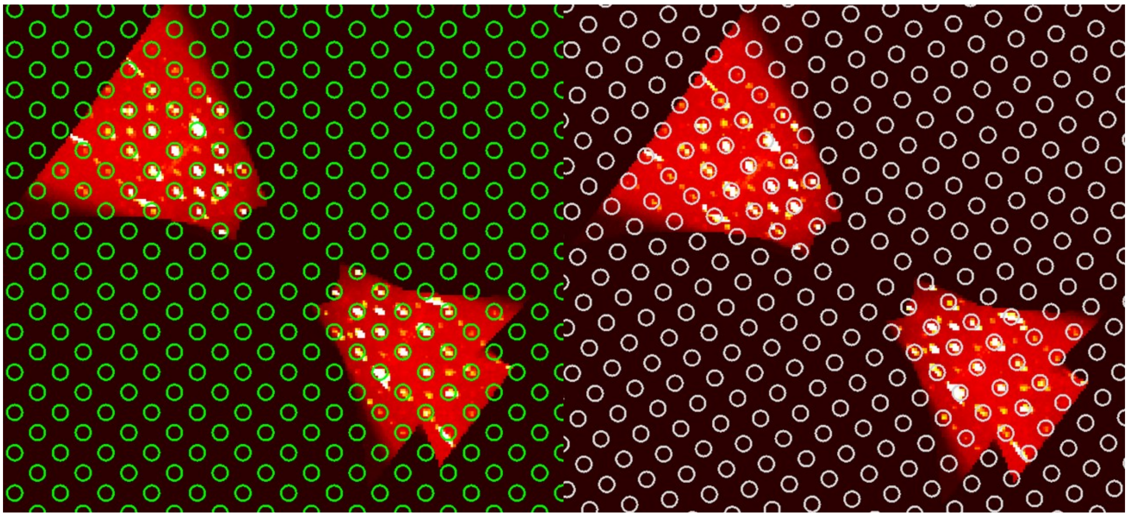

Figure S5: Framework, **1**, showing hk0 synthetic precession image. Note the two overlays from the predicted positions for reflections from component 1 (green overlay) and 2 (white overlay) caused by the phase transition.

References

(1) Piermanrini, S. B. G. J., Barnett, J. D., Forman, R. A., *J Appl Phys*, **1975**, *46*, 2774-2780
(2) Bruker, Madison, Wisconsin, USA, 2006
(3) Krause, R. H.-I. L., Sheldrick, D. M., Stalke, D., *J Appl Cryst*, **2013**, *48*, 3-10
(4) Betteridge, P.W., Carruthers, J. R., Cooper, R. I., Prout, K. P., Watkin, D. J., *J Appl Cryst*, **2003**, *36*, 1487
(5) Spek, A. L., *Acta Cryst C*, **2015**, *71*, 9-18
(6) Dawson, A., Parsons, S., Ruf, M., *J Appl Cryst*, **2004**, *37*, 410-416
(7) Parsons, S., 2004
